# Botulinum toxin injection induced autoimmune thyroiditis and myxedema coma: A case report

**DOI:** 10.1177/2050313X261430649

**Published:** 2026-03-31

**Authors:** Khaled Gazwi, Tareq Zaza, Hassan Mitwally, Nikeshkumar Davda, Adel Ganaw

**Affiliations:** 1 Critical Care Department, Al-Wakra Hospital, Hamad Medical Corporation, Doha, Qatar; 2 Pharmacy Department, Al-Wakra Hospital, Hamad Medical Corporation, Doha, Qatar; 3 Anesthesia and Critical Care Department, Al-Wakra Hospital, Hamad Medical Corporation, Doha, Qatar

**Keywords:** hypothyroidism, botulinum toxin, autoimmune thyroiditis, Hashimoto thyroiditis, myxedema coma

## Abstract

This case describes a 28-year-old female with amyotrophic lateral sclerosis and recurrent urinary tract infections who developed myxedema coma following a botulinum toxin type-A (Btx) injection for muscle spasticity. On intensive care unit admission, she presented with hypothermia, bradycardia, and reduced consciousness. Laboratory evaluation revealed markedly elevated thyroid-stimulating hormone levels, confirming myxedema coma. Prompt treatment with intravenous levothyroxine and hydrocortisone resulted in rapid improvement in temperature and mental status. Further investigation showed elevated antithyroid peroxidase antibodies, suggesting drug-induced autoimmune thyroiditis triggered by Btx. This case highlights the potential link between Btx injection and thyroid autoimmunity, emphasizing the importance of early recognition and monitoring of thyroid function to prevent life-threatening complications in at-risk patients.

## Introduction

Spasticity, a hallmark of upper motor neuron disease, is commonly observed in conditions such as cerebral palsy, stroke, multiple sclerosis, and brain injury. It often leads to pain, deformity, and impaired functional ability. Botulinum toxin type-A (Btx) has been established as an effective treatment for focal spasticity.^1,2^ It works by presynaptically blocking transmission at the neuromuscular junction, leading to localized and temporary paresis of the affected muscles.^3^ This intervention not only reduces spasticity but also provides an analgesic effect that can last up to 4 months, thereby improving the range of movement, functional abilities, daily activities, and overall quality of life.^2^

Despite its widespread use and safety profile, there are reports of both local and systemic adverse effects across multiple organ systems, including musculoskeletal, neurological, visual, respiratory, immune, gastrointestinal, urinary, and cardiovascular systems.^3^

Here, we present the case of a 28-year-old female who developed autoimmune thyroiditis and myxedema coma following a Btx injection. To our knowledge, this is the first reported instance of such a critical adverse effect associated with this treatment.

## Case description

A 28-year-old female patient a known case of amyotrophic lateral sclerosis complicated by muscle spasms and contracting joints, necessitating wheelchair use. Her baseline Glasgow Coma Score (GCS) is 11, characterized by spontaneous eye opening, localization to noxious stimuli, and incomprehensive vocalizations (E4 M5 V2). Additionally, she has a history of recurrent hospital admissions secondary to urinary tract infections.

**Figure 1.**
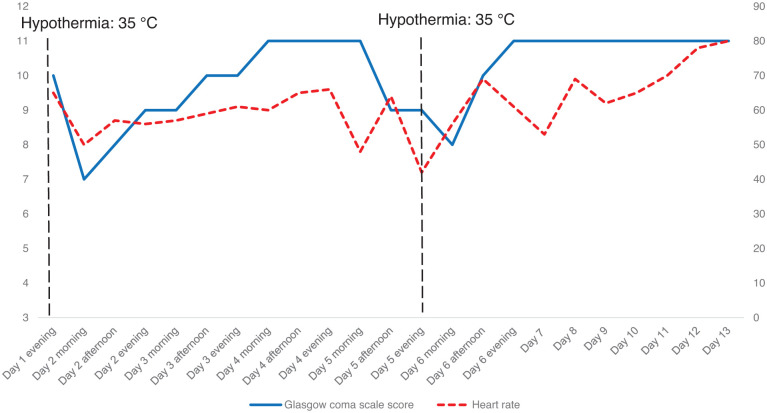
Longitudinal trends of GCS and heart rate during hospitalization. The patient was hypothermic (35°C) on hospital day 1 and 5 (black dotted vertical lines). Solid line: GCS; dashed line: heart rate. GCS: Glasgow Coma Score

On the 8 March 2022, the patient was admitted to the intensive care unit (ICU) at our hospital presenting with hypothermia (35°C), bradycardia with a heart rate of 50 beats per minute (bpm), and a decreased level of consciousness. The patient had a normal leukocyte count (5.5 × 10^9^/L; normal range 4.0–11.0 × 10^9^/L) with low inflammatory markers (C-reactive protein 33 mg/L; normal range <5 mg/L, and procalcitonin 0.15 ng/mL; normal range <0.5 ng/mL). Notably, 3 days prior to admission, the patient sought medical attention at a private hospital due to increased lethargy as reported by her family. A computed tomography scan was performed, showing a normal study. Subsequently, a thyroid-stimulating hormone (TSH) test was conducted, revealing a critically high level of 125 mIU/L (normal range: 0.45–4.5 mIU/L). Treatment was initiated with levothyroxine at a dosage of 50 mcg daily, with two doses administered before the patient’s admission to our hospital. Upon admission, a repeat thyroid profile was obtained, demonstrating a persistently elevated TSH level of 167 mIU/L, accompanied by an unrecordable free T4 level below 3.2 pmol/L (normal range: 20–90 pmol/L). Based on the patient’s clinical signs and symptoms, along with laboratory findings indicative of severe hypothyroidism, a diagnosis of myxedema coma was confirmed. Treatment was initiated with intravenous hydrocortisone 50 mg every 6 h and a total of 200 mcg of intravenous levothyroxine administered on day 1. Subsequent management included the administration of 100 mcg of intravenous thyroxine on both day 2 and 3. On ICU admission day 2, the patient’s body temperature normalized, accompanied by a slight improvement in consciousness level (refer to Figure 1). On day 4 the patient was normothermic and hemodynamically stable, her heart rate ranges from 60 to 70 bpm, and she returned almost to her baseline consciousness, GCS 11 (Figure 1). Subsequently, the intravenous levothyroxine was transitioned to an oral dosage of 100 mcg daily, starting from day 4. Concurrently, hydrocortisone was discontinued.

Upon further inquiry into the patient’s medical history, her family reported that she had received a Btx injection 3 months prior to the current presentation, for the management of her spastic muscles. Notably, the patient had a documented normal thyroid profile 1 month before the Btx injection with TSH level 3.41 mIU/L and free thyroxine (FT4) levels 11.3 pmol/L. The family denied any history of viral infections or influenza during the aforementioned period. Given the temporal relationship between the Btx injection and the onset of thyroid dysfunction, drug-induced autoimmune thyroiditis was suspected. Subsequent testing for anti-thyroid peroxidase antibodies revealed elevated levels of 123 IU/mL (normal range: <35 IU/mL), further supporting the diagnosis.

On day 5, the patient was transferred out of the ICU. However, a rapid response team alarm was triggered later that day due to a seizure episode, accompanied by hypothermia (35°C) and bradycardia, with a heart rate dropping to 40 bpm. Consequently, the patient was promptly transferred back to the ICU and treatment with intravenous levothyroxine and hydrocortisone at a dosage of 50 mg every 6 h was reinstated for a duration of 4 days. By day 7, the patient’s condition had stabilized, and she had returned to her baseline status (Figure 1). On day 9, hydrocortisone was discontinued, and the patient’s levothyroxine regimen was transitioned to oral administration. Subsequently, on day 13, the patient transferred out of the ICU to the medical ward. On hospital day 15, the patient was discharged home on levothyroxine 100 mcg daily with endocrinology follow-up.

## Discussion

Chronic autoimmune thyroiditis (Hashimoto’s thyroiditis) is the most common cause of hypothyroidism in iodine-sufficient areas of the world.^4^ As reflected by the name, it is autoimmune-mediated destruction of the thyroid gland involving apoptosis of thyroid epithelial cells. On histopathology, it is characterized by profuse lymphocytic infiltration, lymphoid germinal centers, and destruction of thyroid follicles.^5^ There are several proposed theories for the potential mechanism of this autoimmune response leading to thyroid destruction, one of those theories is “molecular mimicry.” According to this theory, there is an immune response to a foreign antigen that is structurally similar to an endogenous substance.^6^

Gregoric et al. reported a case with an analysis of amino acid sequence for the Clostridium Btx molecule comparing it to the thyroid autoantigen molecule. They found that (i) Btx and thyroid autoantigens share amino acid sequence homology; (ii) some homologous regions contain epitopes of both Btx and thyroid autoantigens; and (iii) some of such regions contain HLA-DR3 and/or HLA-DR7 binding motifs, which predominate over other HLA-DRs.^5^ This has led to their conclusion that there is clinical and bioinformatics data suggesting a possible pathogenetic link between Clostridium Btx injection and autoimmune thyroid diseases.

Our case had a chronological sequence of events consistent with the possible connection between Btx injection and the development of thyroiditis as detailed in the case presentation section. The patient also had no viral illness or other incident that could be a trigger for the immune response activation that leads to thyroiditis during the 4 months prior to her presentation. As noted in the presentation section, the patient had normal thyroid function 4 months prior to this incident. The calculated Naranjo adverse drug reaction scale^7^ is 5 which reveals a probable correlation between Btx and autoimmune thyroiditis in this case (Table 1).

**Table 1.** Naranjo adverse drug reaction probability scale. Assessment of the likelihood of an adverse drug reaction using the Naranjo Adverse Drug Reaction Probability Scale. The patient scored 5, indicating a probable relationship between botulinum toxin type-A (Btx) injection and the development of autoimmune thyroiditis/myxedema coma.

Question	Yes	No	Don’t know	Score
1. Are there previous conclusive reports on this reaction?	+1	0	0	0
2. Did adverse event appear after the suspected drug was given?	+2	−1	0	+2
3. Did the adverse reaction improve when the drug was discontinued or a specific antagonist was given?	+1	0	0	0
4. Did the adverse reaction appear when the drug was readministered?	+2	−1	0	0
5. Are there alternative causes that could have caused the reaction?	−1	+2	0	+2
6. Did the reaction reappear when a placebo was given?	−1	+1	0	0
7. Was the drug detected in any body fluid in toxic concentrations?	+1	0	0	0
8. Was the reaction more severe when the dose was increased, or less severe when the dose was decreased?	+1	0	0	0
9. Did the patient have a similar reaction to the same or similar drugs in any previous exposure?	+1	0	0	0
10. Was the adverse event confirmed by any objective evidence?	+1	0	0	+1
Total score				5^a^ (probable)
Total score	Interpretation			
⩾9	Definite			
5–8	Probable			
1–4	Possible			
⩽0	Doubtful			

a The adverse drug reaction probability is based on the total score.

Based on our findings, we share the same conclusion as Gregoric et al. that Btx could be a trigger for autoimmune thyroiditis. The diagnosis could be missed or delayed due to the frequently observed subclinical course of autoimmune thyroid diseases. Our aim by reporting this case is to increase awareness about the possible connection between Btx injection and the development of autoimmune thyroiditis, which could remain subclinical for a while. Because symptoms of hypothyroidism could be nonspecific, this awareness may help clinicians maintain a certain level of suspicion, lead to early detection of the disease, and avoiding a delay in making diagnosis until the patient presents with what could be a life-threatening condition like our case.

## Conclusions

In conclusion, our case underscores the potential link between Btx administration and the development of autoimmune thyroiditis. Given the increasing use of Btx in medical and cosmetic procedures, clinicians must remain vigilant regarding this adverse drug reaction. When evaluating patients presenting with thyroid dysfunction following Btx treatment, clinicians should consider autoimmune thyroiditis as a differential diagnosis, particularly after excluding other possible causes. Heightened awareness of this drug interaction is essential for ensuring timely diagnosis and appropriate management, thereby optimizing patient outcomes and safety. Further research and larger-scale studies are warranted to better understand the underlying mechanisms and risk factors associated with Btx-induced autoimmune thyroiditis.

## References

[1] Shaw L Rodgers H Price C , et al. BoTULS: a multicentre randomised controlled trial to evaluate the clinical effectiveness and cost-effectiveness of treating upper limb spasticity due to stroke with botulinum toxin type A. Health Technol Assess 2010; 14(26): 1–113. 10.3310/hta14260 20515600

[2] O’Brien CF. Treatment of spasticity with botulinum toxin. Clin J Pain 2002; 18(6 Suppl): S182–S190. 10.1097/00002508-200211001-00011 12569967

[3] Phadke CP On AY Kirazli Y , et al. Adverse clinical effects of botulinum toxin intramuscular injections for spasticity. Can J Neurol Sci 2016; 43(2): 298–310. 26597813 10.1017/cjn.2015.314

[4] Hollowell JG. Serum TSH, T4, and thyroid antibodies in the United States population (1988 to 1994): National Health and Nutrition Examination Survey (NHANES III). J Clin Endocrinol Metab 2002; 87(2): 489–499. 11836274 10.1210/jcem.87.2.8182

[5] Gregoric E Gregoric JA Guarneri F , et al. Injections of *Clostridium botulinum* neurotoxin A may cause thyroid complications in predisposed persons based on molecular mimicry with thyroid autoantigens. Endocrine 2011; 39(1): 13–20. 21061092 10.1007/s12020-010-9410-9

[6] Rojas M Restrepo-Jiménez P Monsalve DM , et al. Molecular mimicry and autoimmunity. J Autoimmun 2018; 95: 100–123. 30509385 10.1016/j.jaut.2018.10.012

[7] Naranjo CA Busto U Sellers EM , et al. A method for estimating the probability of adverse drug reactions. Clin Pharmacol Ther 1981; 30(2): 239–245. 7249508 10.1038/clpt.1981.154

